# Molecular investigations of cat fleas (*Ctenocephalides felis*) provide the first evidence of *Rickettsia felis* in Malta and *Candidatus* Rickettsia senegalensis in Israel

**DOI:** 10.1016/j.nmni.2018.05.001

**Published:** 2018-05-22

**Authors:** S. Hornok, G. Baneth, A. Grima, N. Takács, J. Kontschán, M.L. Meli, V. Suter, H. Salant, R. Farkas, R. Hofmann-Lehmann

**Affiliations:** 1) Department of Parasitology and Zoology, University of Veterinary Medicine, Budapest, Hungary; 2) Koret School of Veterinary Medicine, Hebrew University, Yehoshua Hankin, Israel; 3) Plant Protection Institute, Centre for Agricultural Research, Hungarian Academy of Sciences, Budapest, Hungary; 4) Clinical Laboratory and Center for Clinical Studies, Vetsuisse Faculty, University of Zurich, Zurich, Switzerland

**Keywords:** Emerging, *gltA* gene, *ompA* gene, phylogeny, *Rickettsia*, 17 kDa protein gene

## Abstract

*Rickettsia felis*, the causative agent of flea-borne spotted fever, occurs on all continents except Antarctica, owing to the cosmopolitan distribution of its cat flea vector. In this study, cat fleas were collected in two countries where the occurrence of *R. felis* was either unknown (Malta) or where accurate prevalence data were lacking (Israel). Altogether 129 fleas were molecularly analysed for the presence of rickettsial DNA. On the basis of three genetic markers, *R. felis* was identified in 39.5% (15/38) of the cat fleas from Malta. Sequences showed 100% identity to each other and to relevant sequences in GenBank. Among the 91 cat fleas from Israel, two (2.2%) contained the DNA of *Candidatus* Rickettsia senegalensis. Phylogenetically, the *R. felis* and *Candidatus* R. senegalensis identified here clustered separately (with high support) but within one clade, which was a sister group to that formed by the typhus group and spotted fever group rickettsiae. This is the first record of *R. felis* in Malta and of *Candidatus* R. senegalensis outside its formerly reported geographical range including Africa, Asia and North America.

## Introduction

Rickettsiae are obligate intracellular Gram-negative bacteria which may affect their vertebrate hosts after arthropod-borne transmission [1]. Thus, the life cycle of pathogenic rickettsiae necessitates the presence of a blood-sucking vector [2]. The primary arthropod vectors and reservoirs vary according to *Rickettsia* spp., i.e. *R. prowazekii* and *R. typhi* in the typhus group (TG) are louse and flea borne [3], whereas nearly 20 *Rickettsia* species in the spotted fever group (SFG) are mite and tick borne [4]. In addition, *R. felis*, the causative agent of flea-borne spotted fever, is transmitted by the cat flea (*Ctenocephalides felis*), but it has also been demonstrated from a broad range of arthropods [2], [5].

During the last decade, *R. felis*–like organisms (RFLOs) have been identified with molecular methods in various arthropods, including cat fleas [5]. Among these, there are genetic variants, which (on the basis of their sequence divergence) have been proposed as new species, as exemplified by *Rickettsia asembonensis* [6] and *Candidatus* Rickettsia senegalensis [2]. The geographical distribution of RFLOs appears to be broad in a worldwide context, but their pathogenicity is unknown [7]. The sympatric occurrence of *R. felis* and RFLOs has also been reported [5], [8]. However, the role of RFLOs in modulating the vertical and horizontal transmission of other sympatric rickettsiae remains to be clarified [7].

Similar to RFLOs, *R. felis* occurs on all continents except Antarctica, owing to the cosmopolitan distribution of its vector, the cat flea [5]. During the past 15 years, approximately 30 countries were put on the map of flea-borne spotted fever [9], but there are regions without relevant information. The latter is exemplified by the middle and eastern regions of the Mediterranean Basin, where in several countries (including Malta) the occurrence of *R. felis* is unknown or actual/updated prevalence data are lacking (e.g. Israel; reported pool prevalence [10]). Therefore, in this study, cat fleas from Malta and Israel were molecularly analysed for the presence of rickettsial DNA.

## Materials and methods

During the study, the following numbers of cat fleas were collected in 2017: 38 from 11 cats and three dogs at eight locations (data not shown) in Malta, and 91 specimens from 28 cats and three dogs in Jerusalem, Israel. Fleas were removed from these animals during regular veterinary care; therefore, no ethical permission was needed. Fleas from each host were stored in 96% ethanol separately, and their species was identified according to Whitaker [11].

DNA was extracted from individual fleas with the QIAamp DNA Mini Kit (Qiagen, Hilden, Germany) following the manufacturer's instructions, including an overnight digestion in tissue lysis buffer and proteinase K at 56°C. Extraction controls were used to monitor cross-contamination among samples. All samples were tested for the quantity and quality of DNA contents with a TaqMan real-time PCR specific for the 18S rRNA gene (Thermo Fisher Scientific, Vantaa, Finland) [12].

Flea DNA extracts were screened for the presence of rickettsiae with a TaqMan PCR amplifying a 74 bp fragment of the citrate synthase (*gltA*) gene of SFG and TG rickettsiae [12]. From the positive samples, a 796 bp long fragment of the *gltA* gene was amplified for sequencing with primers CS477f and CS1273r [13], as previously described [12]. In addition, an approximately 480 bp long fragment of the 17 kDa surface antigen gene of *Rickettsia* spp. was amplified with primers 17kd1 (5′-GCT CTT GCA ACT TCT ATG TT-3′) and 17kd2 (5′-CAT TGT TCG TCA GGT TGG CG-3′) [14]. The 25.0 μL final volume of reaction mixture contained 5.0 μL template DNA, 0.5 U HotStar Taq Plus DNA Polymerase (5 U/μL) (Qiagen), 2.5 μL of 10 × Coral Load PCR buffer (15 mM MgCl_2_ included), 0.5 μL dNTP Mix (10 mM), 0.5 μL of each primer (50 μM) and 15.9 μL distilled water. The thermal cycle included an initial denaturation step at 95°C for 5 minutes, followed by 40 cycles of denaturation at 95°C for 30 seconds, annealing at 51°C for 30 seconds and extension at 72°C for 1 minute. Final extension was performed at 72°C for 5 minutes. In a fourth PCR, an approximately 532 bp long fragment of the outer membrane protein A (*ompA*) gene of *Rickettsia* spp. was amplified with primers Rr190.70p (5′-ATG GCG AAT ATT TCT CCA AAA-3′) and Rr190.602n (5′-AGT GCA GCA TTC GCT CCC CCT-3′) [15]. Conditions were the same as above, except using 1.0 U polymerase and annealing at 48°C for 30 seconds.

PCR products of the 17 kDa and *ompA* genes were sequenced at Biomi (Gödöllő, Hungary) and those of the *gltA* gene at Microsynth (Balgach, Switzerland). Sequences were edited and assembled using Geneious 9.1.7 (http://www.geneious.com [16]), then aligned and compared to reference GenBank sequences by the nucleotide BLASTn programme (https://blast.ncbi.nlm.nih.gov). Representative sequences were submitted to GenBank (*R. felis* from Malta: MG893575 (*gltA* gene), MG893577 (17 kDa antigen gene), MG893579 (*ompA* gene); *Candidatus* R. senegalensis from Israel: MG893576 (*gltA* gene), MG893578 (17 kDa antigen gene)). Phylogenetic analyses were performed by the maximum-likelihood method and Tamura3 model using MEGA 6.0. Exact confidence intervals for the prevalence rates were calculated at the 95% level.

## Results

Among the 38 DNA extracts of cat fleas from Malta, 15 (39.5%; 95% confidence interval, 24–56.6) were *gltA* PCR positive for rickettsiae. In all of these samples, *R. felis* was identified by sequencing, with 100% (757/757 bp) identity to each other and to *R. felis* sequences in GenBank (JQ674484 from Gabon, AF210692 from the United States). The amplified parts of the 17 kDa and *ompA* genes were also 100% (385/385 bp and 452/452 bp, respectively) identical with those of *R. felis* (e.g. KF241853 and AJ563398, respectively, from Mexico).

Among the 91 DNA extracts of cat fleas from Israel, two (2.2%; 95% confidence interval, 0.3–7.7) were *gltA* PCR positive for rickettsiae. In both of these samples *Candidatus* R. senegalensis was identified by sequencing, with 100% identity to each other and to two conspecific sequences in GenBank (757/757 bp identity with KF666472 from Senegal, 736/736 bp identity with KU499847 from India). The amplified part of the 17 kDa gene was also 100% (385/385 and 375/375 bp) identical with that of *Candidatus* R. senegalensis reported from the United States (AY953285 and KU167051-2, respectively). Amplification of the *ompA* gene was not successful from these samples.

**Fig. 1:**
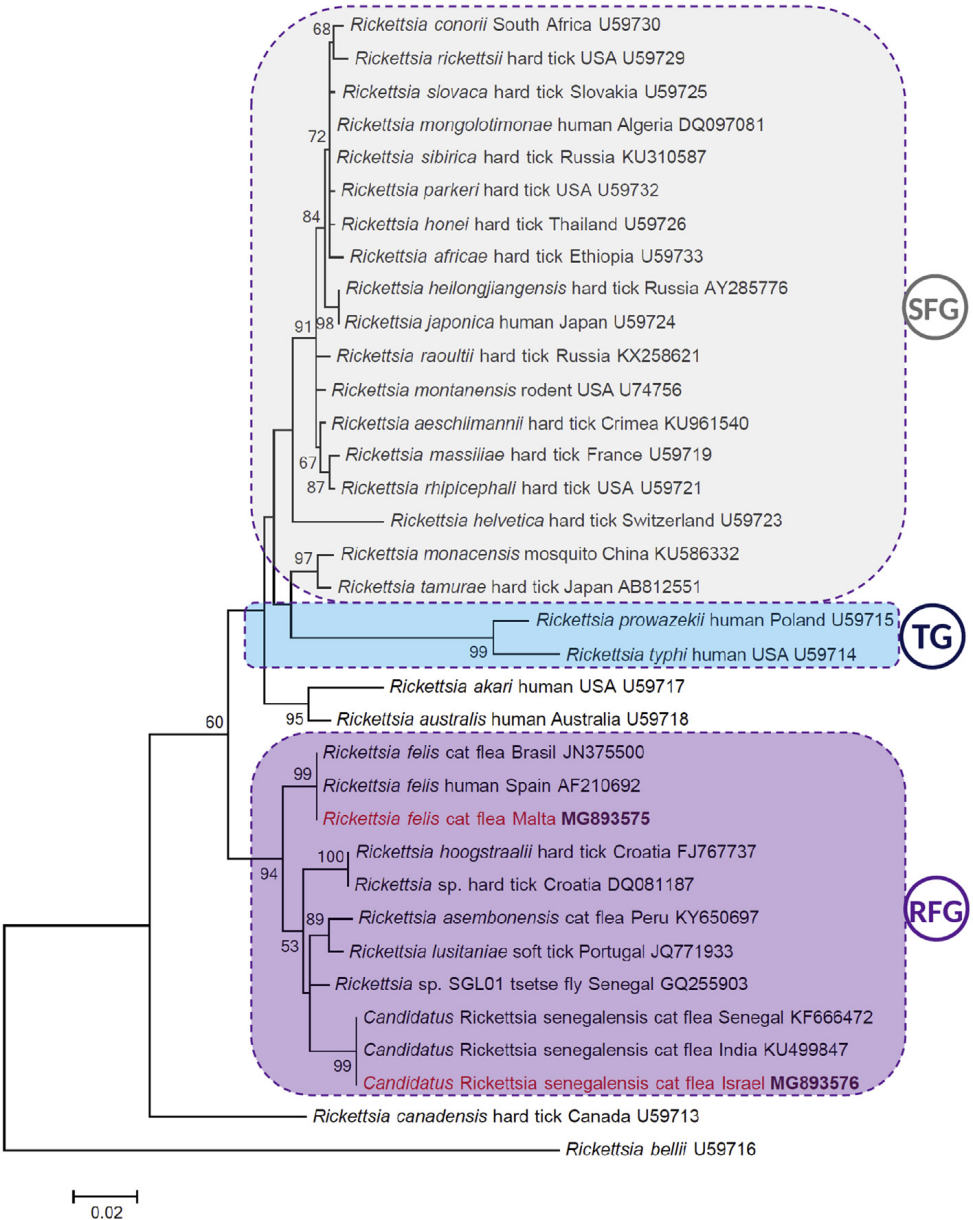
Maximum-likelihood tree of *Rickettsia* spp. based on *gltA* sequences. Two sequences identified in this study are shown in red with bold accession numbers. Further sequences of *Rickettsia* spp. (representing major phylogenetic groups and having nearly 100% coverage with sequences obtained here) were retrieved from GenBank. Clades of RFG (*Rickettsia felis* group), SFG (spotted fever group) and TG (typhus group) are circled by dashed line filled with different colours. Species name, isolation source and country of origin are shown for each entry. Branch lengths represent number of substitutions per site, inferred according to scale shown.

Phylogenetically, *R. felis* and *Candidatus* R. senegalensis identified here clustered separately (with 94% bootstrap support) but within one clade, which was a sister group to that formed by TG and SFG rickettsiae (Fig. 1).

## Discussion

Epidemiologic studies of *R. felis* and related bacteria are considered to be important because their natural cycles have not yet been fully elucidated [2]. The recent emergence of *R. felis*–associated febrile diseases in West and East Africa further justifies this [2]. While clinical cases of *R. felis* infection in humans are well documented, the pathogenicity of *Candidatus* R. senegalensis is unknown. However, a *Rickettsia* species with similar sequence (with 98.5%, i.e. 673/683 bp, identity) has been reported in Senegal from a human patient with febrile illness (JQ674485) [2].

Assessing the significance of the present findings in a geographical context, the first record of *R. felis* in Malta provides new information on the country level and suggests a relatively high epidemiologic risk of human infection in that island. For comparison, 25.8% of cat fleas were shown to carry *R. felis* in Sicily, southern Italy [17], which is a lower prevalence rate compared to the 39.5% found here.

However, the first detection of *Candidatus* R. senegalensis in Israel is new on the continental level because this species has been hitherto reported at great distances from the Middle East, i.e. in Africa (Senegal, KF666472), Asia (India, KU499847) and North America (United States, AY953285, KU167051). In addition, a *Rickettsia* genotype which has highly similar *gltA* sequence to *Candidatus* R. senegalensis was identified in Asia (Thailand, AF516331) and a further one with a similar sequence in Africa (Ivory Coast, JN620082; Gabon, JQ354961). At the same time, the *gltA* sequence identified here in cat fleas from Israel was only 96% (328/340 bp) similar to an RFLO (under accession no. KP050777) formerly identified in desert fleas in Israel [18].

Phylogenetic analyses of *R. felis* and *Candidatus* R. senegalensis were performed in a previous study [2]. On the basis of the *gltA* gene, it was established that these two species, together with *Rickettsia hoogstraalii*, form a compact, isolated clade that is a sister group to the neighbouring *Rickettsia akari* and *Rickettsia australis* cluster [2]. However, in that phylogenetic analysis, SFG rickettsiae appeared to be underrepresented. By including a higher number of SFG *Rickettsia* spp. in the phylogenetic analysis here, not only was the separateness of the *R. felis* clade confirmed, but this also became a sister group to the cluster containing all SFG and TG species, as well as *R. akari* and *R. australis* (Fig. 1). This pattern was confirmed in maximum-likelihood analyses with other models (Hasegawa-Kishino-Yano and Tamura-Nei; tree not shown). Therefore, inclusion of the *R. felis* cluster in phylogenetic trees [2] argues against the formerly reported basal position of the TG among rickettsiae [1].

This pattern of phylogenetic clustering of *R. felis* and closely related species or genotypes was confirmed with other molecular markers [2] except *ompA.* Concerning the latter, it has been reported that the amplification of the *ompA* gene typical for the SFG of rickettsiae was unsuccessful in the case of *Candidatus* R. senegalensis [2], similar to what was experienced in this study.

In conclusion, the results of the present study broaden the geographical range of *R. felis* and *Candidatus* R. senegalensis. These findings also prompt extension of molecular analyses of cat fleas (the flea species with the most cosmopolitan distribution) for rickettsiae in those countries, where relevant data are lacking.
